# Elevated Procalcitonin in Acute Pseudogout Flare: A Case Report

**DOI:** 10.7759/cureus.4853

**Published:** 2019-06-06

**Authors:** Shilpa Vasishta, Satya Patel

**Affiliations:** 1 Internal Medicine, University of California Los Angeles, Los Angeles, USA

**Keywords:** procalcitonin, pseudogout, cppd

## Abstract

An 86-year-old male presented to the emergency department with fevers and tachycardia. Given concern for sepsis, a broad infectious workup was pursued. Though no source of infection was identified, the patient was found to have an elevated procalcitonin level. The patient subsequently developed painful joint effusions of the knees, which on arthrocentesis were consistent with calcium pyrophosphate deposition (CPPD) or “pseudogout”. His symptoms of fevers and arthralgias resolved with anti-inflammatory agents. CPPD is a known cause of systemic inflammatory response syndrome (SIRS) in elderly patients. Procalcitonin has been studied as a biomarker for distinguishing infectious from non-infectious causes of SIRS, although its use in CPPD and other crystal arthropathies is not well-defined. The current case is the first to describe elevated procalcitonin specifically attributable to acute pseudogout flare and highlights the need for further study of this biomarker in non-infectious, pro-inflammatory states.

## Introduction

Calcium pyrophosphate deposition (CPPD) disease is a common cause of inflammatory arthritis in patients of advanced age [1]. CPPD flares may present with fever, leukocytosis, and other signs of systemic inflammation that mimic acute infection [2-4]. Therefore, researchers have worked to identify serum markers that may reliably distinguish infection from other pro-inflammatory states. Procalcitonin has been identified in certain clinical contexts as a specific marker for acute bacterial infection, and studies to date suggest a promising role for its use in distinguishing infection from other inflammatory states [5-14]. However, studies also suggest potential limitations in the use of procalcitonin [11-16]. Herein, we report a case of systemic inflammatory response syndrome (SIRS) with elevated procalcitonin ultimately attributed to a CPPD flare. Our case highlights the need for further studies of procalcitonin in patients with CPPD and other inflammatory arthropathies to guide appropriate use of this biomarker.

## Case presentation

An 86-year-old male with a history of stroke and osteoarthritis was admitted for high fevers and tachycardia concerning for sepsis. The patient had no localizing infectious symptoms such as cough, abdominal pain, or dysuria. Physical exam was notable for a temperature of 104 °F and a heart rate of 120 beats per minute; the remainder of his vital signs and exam were unremarkable. Laboratory assessment results were notable, with white blood cell (WBC) count being 9,000 per uL (normal range: 4,500 to 11,000 per uL), lactate 1.1 mmol/L (normal range: 0.5 to 2.2 mmol/L), and procalcitonin 0.97 ng/mL (normal: <0.2 ng/mL). The patient was resuscitated with intravenous fluids and treated empirically with levofloxacin and metronidazole, given the multiple risk factors for aspiration pneumonia. 

Over 72 hours, high fever and tachycardia persisted in the patient despite broad antibiotic coverage, and he continued to have no localizing signs or symptoms of infection. Computed tomography (CT) of the chest, abdomen, and pelvis revealed no occult infectious source and blood cultures remained negative, though procalcitonin continued to increase to 1.99 ng/mL. 

**Figure 1:**
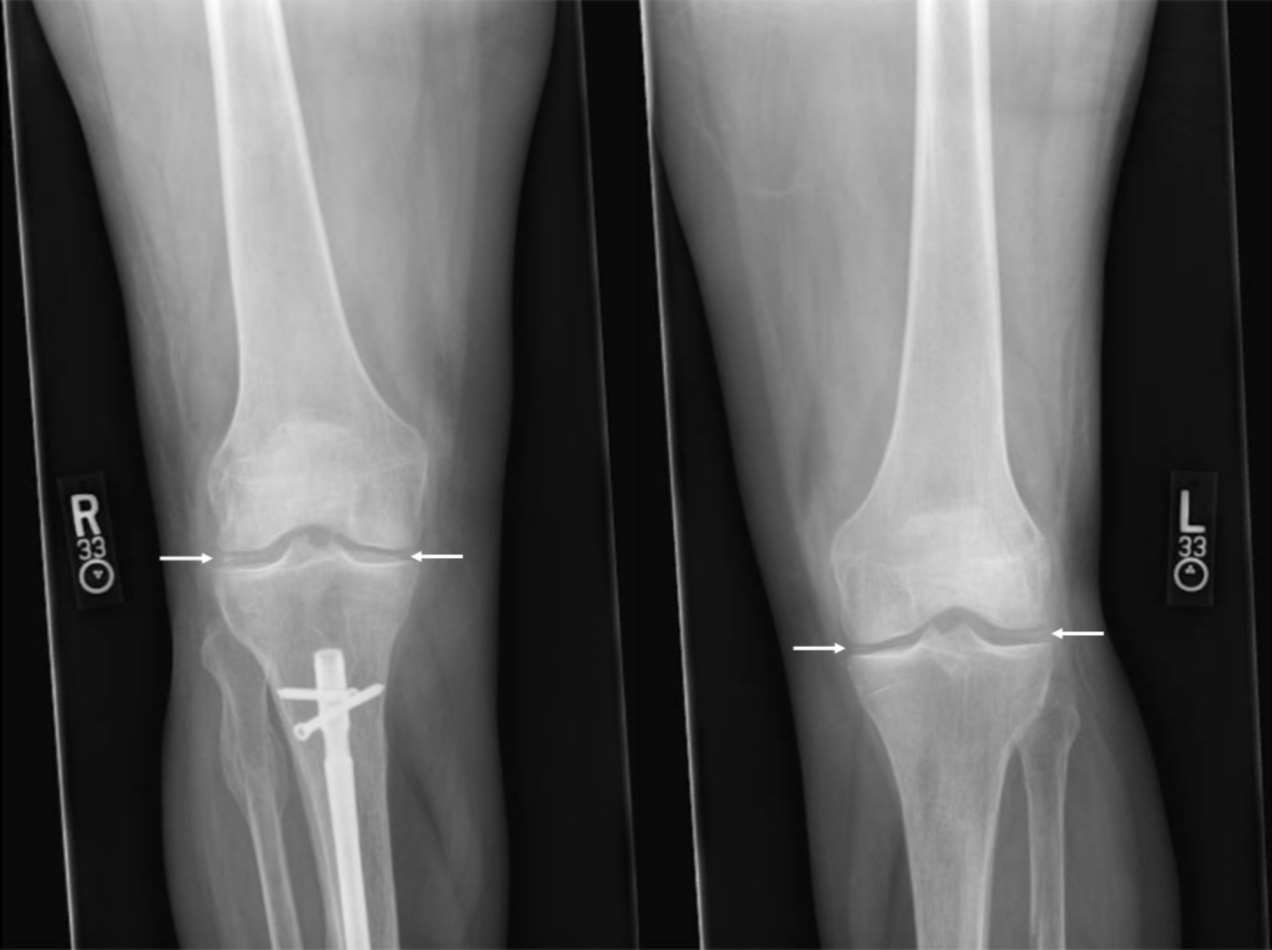
Chondrocalcinosis of bilateral knees

On hospital day 4, the patient noted new pain and swelling of both knees, and physical exam demonstrated new bilateral suprapatellar effusions. X-ray of the knee joints revealed bilateral chondrocalcinosis (Figure 1). Arthrocentesis of the left knee yielded cloudy yellow synovial fluid with 15,000 WBCs per uL (normal range 0 to 200 per uL), 90% neutrophils (normal range 0 to 25%) and negative gram stain and culture consistent with aseptic, inflammatory arthritis. Microscopy demonstrated calcium pyrophosphate crystals consistent with CPPD. The patient was thus diagnosed with acute pseudogout flare and treated with colchicine; antibiotics were discontinued. Fever and tachycardia improved over 48 hours and the patient was discharged in stable condition. 

## Discussion

CPPD is inflammatory crystal arthritis caused by deposition of calcium pyrophosphate in the synovial space. The condition presents as acute mono- or polyarticular flares of pain and swelling involving the large joints and most commonly the knees. CPPD is most prevalent in patients over 60 years of age and in those with underlying osteoarthritis [1].

CPPD flares may present acutely with fevers, tachycardia and other manifestations of systemic inflammation, which in some cases precede localizing symptoms such as joint pain or swelling [2-4]. The diagnosis of CPPD requires a high index of suspicion and thorough infectious evaluation, which may include arthrocentesis for assessment of superimposed septic arthritis [5-7].

Procalcitonin is an inflammatory marker that has been recognized for its utility in distinguishing infectious from non-infectious causes of systemic inflammation. Prior studies have found that a procalcitonin level above 0.5 ng/mL may reliably predict infection in patients with underlying inflammatory conditions, with reported specificity ranging from 81% to 98% [5-14]. However, studies to date also highlight clinical scenarios in which procalcitonin levels must be interpreted with caution. For example, in antineutrophil cytoplasmic antibodies (ANCA) associated vasculitis, the use of procalcitonin may require an adjusted ‘normal range’ to reliably identify underlying infection [11-12]. Additionally, in adult-onset Still's disease and acute gout flares, procalcitonin may be less reliable as an infectious marker [13-16]. Whether these findings may be reproduced in larger studies or have application in acute pseudogout flares remains unknown. 

To our knowledge, this is the first reported case of elevated procalcitonin specifically attributable to acute CPPD flare. Further study is warranted to characterize the utility of procalcitonin in identifying infection among patients with CPPD and related inflammatory arthropathies.

## Conclusions

The current report describes a case of acute CPPD flare that was initially misdiagnosed as sepsis due to multiple suggestive features including elevated procalcitonin. This case adds to a growing body of literature describing the complexities of interpreting procalcitonin in inflammatory states. While procalcitonin has shown promise as a marker of infection in patients with underlying inflammatory conditions, important limitations continue to emerge in its use across a range of disease states and clinical contexts. Further study is warranted to delineate the appropriate use of procalcitonin in patients with inflammatory conditions including crystal arthropathies.
